# DFT Study of Americium and Europium Complexation with 2,9-Bis(1,2,4-triazin-3-yl)-1,10-Phenanthroline Ligand: The Influence of the Counteranions–Nitrate Versus Perchlorate

**DOI:** 10.3390/molecules31040665

**Published:** 2026-02-14

**Authors:** Jaanus Burk, Lauri Sikk, Kaido Tämm, Peeter Burk

**Affiliations:** Institute of Chemistry, University of Tartu, Ravila 14a, 50411 Tartu, Estonia; jaanus.burk@gmail.com (J.B.); kaido.tamm@ut.ee (K.T.)

**Keywords:** europium, americium, BTPhen, complexation, DFT

## Abstract

2,9-Bis(1,2,4-triazin-3-yl)-1,10-phenanthroline (BTPhen)-based ligands show great promise in the separation of trivalent lanthanides and actinides. Experimental studies have shown that americium forms stronger complexes with the BTPhen ligands than europium; most theoretical studies have so far failed to reproduce these results. In the current study, three different metal forms (the naked cation, its nitrate or perchlorate salts and tetraaqua solvated salts) were used to study different complexation reactions. It was shown that in the case of naked cations and salts, europium forms the most stable complex with the 2,9-bis(1,2-triazin-3-yl)-1,10-phenanthroline ligand in all of the reactions compared. However, europium is also more strongly interacting (compared to americium) with anions and water molecules in the tetraaquatrinitrato or tetraaquatriperchlorato complexes. That shifts the energies of reactions like Am(NO_3_)_3_·4H_2_O + [Eu(H_2_O)_4_BTPhen]^3+^ = [Am(H_2_O)_4_BTPhen]^3+^ + Eu(NO_3_)_3_·4H_2_O in favor of the americium being complexed with BTPhen and europium with anions and water. Therefore, the americium complexes with BTPhen become the more stable form, in an agreement with the experimental studies. Comparison of counterion influence (nitrate vs. perchlorate) indicates that bigger preference for americium over europium complexation corresponds to the nitrate complexes and stems mainly from the fact that in M(NO_3_)_3_(H_2_O)_4_ europium is stabilized more than in M(ClO_4_)_3_(H_2_O)_4_.

## 1. Introduction

One of the main concerns when considering nuclear power programs for energy production is how to handle the highly radioactive and radiotoxic minor actinides. One of the strategies that is heavily researched is Partitioning and Transmutation (P&T), where nuclear fission is used after separating the radioactive minor actinides from the rest of the spent nuclear fuel to produce less radiotoxic elements [1,2].

The nuclear waste reprocessing usually starts with the plutonium and uranium extraction (PUREX) process [3,4]. Next, the diamide extraction (DIAMEX) process [5,6] is carried out, where oxygen-based diglycolamide ligands are used to separate minor actinides and lanthanides from the rest of the spent fuel. The next proposed step would be the selective actinide extraction (SANEX) process [7], after which transmutation of the minor actinides could be carried out. This is key step for the P&T strategy, because lanthanides have higher neutron capture cross-section areas compared to the actinides and will therefore hinder the transmutation [8,9]. Separating the highly radioactive minor actinides from the nonradioactive lanthanides is complicated, due to their similar chemical properties [10,11]. Fortunately, soft N- and S-donor ligands have been shown to separate trivalent actinides effectively from trivalent lanthanides as the bonding between soft ligands and minor actinide is more covalent [12]. This discovery has encouraged decades worth of research [13,14,15,16,17,18] on designing new ligands that could be used to separate minor actinides from lanthanides.

**Figure 1:**
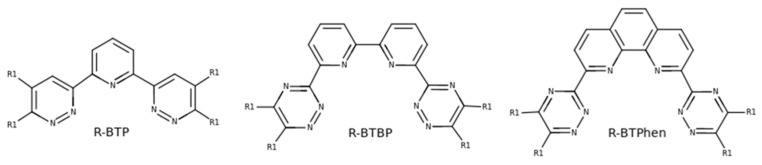
Chemical structures of the three most promising ligand families for SANEX.

Nitrogen containing heteroaromatic moieties have shown the most promise in lanthanide-actinide extractions. In Figure 1 the most promising ligand candidates are shown. Bis(1,2,4-triazin-3-yl)pyridine (BTP) derivatives, designed by Kolarik et al. [19,20], were one of the first ligands that showed high selectivity towards trivalent actinides, but these have found limited use due to difficulties with back-extraction from the organic phase [17]. Drew et al. [21] synthesized the 6,6′-bis(1,2,4-triazin-3-yl)-2,2′-bipyridine (BTBP) ligand, which does not have problems with back-extraction. A major drawback of these ligands is the slow extraction kinetics, which requires the use of phase transfer agents [22]. A major breakthrough was achieved with the synthesis of 2,9-bis(1,2,4-triazin-3-yl)-1,10-phenanthroline (BTPhen) [17,23] that showed good stability towards acidic hydrolysis and exhibited faster extraction kinetics without the need of phase transfer agents.

The rigid structure of the BTPhen ligand simplifies the in silico analysis of the complexation process that helps shed some light on the thermodynamics, bonding characteristics and substituent effects that occur during complex formations in the gas phase and in different media. Xiao et al. [24] studied americium and europium extraction with the BTPhen ligand using B3LYP with the Dolg RECP/6-311G(d,p) basis set. They found that in the gas phase europium formed the more stable [M(BTPhen)]^3+^ complex, which is opposite to what the experiments suggest. However, when they added solvent effects to their calculations, they found that americium gets a bigger effect and thus becomes the more stable complex. A similar study was carried out by Boda et al. [25], who studied the complexation behavior of both BTPhen and BTBP ligands using scalar relativistic DFT in conjunction with the Born–Haber thermodynamic cycle and COSMO solvation model. The study was carried out by using water as the solvent for nitrate and metal ions and octanol for the ligand and complex molecules. They found that the biggest effect of solvation was for the metal cations, while the effect for ligand and metal complexes was negligible.

In our previous work [26], we explored the effect of nitrate ions in lanthanide-actinide extraction with the 2,9-bis(1,2-diazin-3-yl)-1,10-phenanthroline (BTPhen-CH) ligand. We saw that in aqueous media the formation of M(NO_3_)_3_(H_2_O)_4_ complex (M = Eu or Am) had a bigger stabilizing effect for europium. This resulted in the BTPhen-CH ligand having a more favorable complexation with americium ions and thus explaining the selectivity towards actinide extraction. This initiated our interest to see how other anions and different media would affect the extraction kinetics.

The most studied anion besides the nitrate anion is the perchlorate anion. Lewis et al. [15,27] showed that with the BTBP ligand the presence of nitrate ions resulted in only 1:1 metal to ligand complex forming, while in perchlorate the more hydrophobic 1:2 complex was formed. This would indicate that the presence of perchlorate ions increases the extraction rate more than the presence of nitrate ions. In another study [28] they showed that for the BTPhen ligand the 1:2 complex is present both in nitrate and perchlorate anion containing solutions.

Afsar et al. synthesized a CyMe_4_-BTPhen functionalized silica gel magnetic nanoparticle that selectively extracted Am(III) over Eu(III). In order to probe whether the counterion exerts an influence on the extraction efficiency, the extraction experiments were caried out both in HNO_3_ and HClO_4_. While the values of the separation factor in HNO_3_ were SF_Am/Eu_ = 65 ± 5 and SF_Am/Eu_ > 1300 at 1 M and 4 M HNO_3_, respectively, no significant Am/Eu separation was observed in HClO_4_ at these concentrations. The authors proposed that one of the reasons for such a difference might be the formation of a 1:1 complex (M:BTPhen) and the need to saturate the remaining coordination with counterions and the bidentate nature of nitrate ions [29].

Those observations have inspired us to study computationally the interactions of the BTPhen ligand (R_1_–R_4_ = H, Figure 1) with Am(III) and Eu(III) with and without the presence of nitrate or perchlorate ions, both in 1:1 and 1:2 complexes. Our aim was to explain the above-mentioned experimental fact that the presence of nitrate ions shifts the complexation equilibrium in favor of Am(III) complexed with BTPhen.

## 2. Results and Discussion

We have calculated the complexation energies of BTPhen with Am(III) and Eu(III) cations, both in 1:1 and 1:2 (metal:BTPhen) complexes. We have considered also the saturation of free coordination sites of metal cation by three nitrate or perchlorate ions, or by four water molecules for 1:1 complexes. For 1:2 complexes we have also considered additional nitrate or perchlorate ions attached to the metal cation as seen from X-ray experiments [28]. Alongside free metal cations, we also considered neutral salts (M(NO_3_)_3_) and salts hydrated with four water molecules (M(NO_3_)_3_·4H_2_O) as reactants. For salts, hydrated or not, all possible starting arrangements were considered as starting geometries and the most stable structures were used in the following discussion.

**Figure 2:**
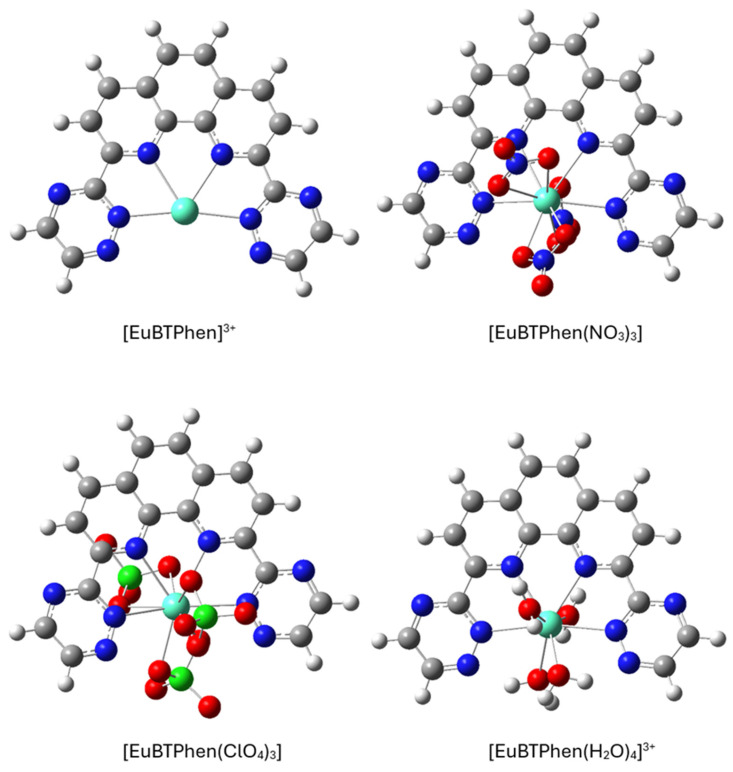
Optimized structures of the europium 1:1 complexes at the PBE1PBE/cc-pVDZ/RECP level of theory.

The 1:1 type of complex occurs when the ligand molecules are not in excess and therefore metal cations can only bind to one ligand molecule. Here we have analyzed three different 1:1 complexes. The first complex contains only the metal cation and BTPhen ligand, in the second form we have added three nitrate or perchlorate ions that make the complex neutral, and in the third four water molecules are added. These additions were chosen based on the knowledge of lanthanides and actinides preferring to be coordinated by 8 to 10 atoms and the fact that these species (NO_3_^−^, ClO_4_^−^, and H_2_O) present in the liquid extraction solutions are relevant to the studied problem. The optimized structures are depicted in Figure 2.

The neutral ligand itself is non-planar as triazine groups are both tilted by 10 degrees relative to the phenanthroline plane so that the triazines are in the parallel arrangement. In complex, the ligand BTPhen is completely planar only in the case of the [M(BTPhen)]^3+^ (M = Eu or Am) complex. In the cases of [M(BTPhen)(NO_3_)_3_], [M(BTPhen)(ClO_4_)_3_], and [M(BTPhen)(H_2_O)_4_]^3+^, the triazine groups are somewhat tilted relative to phenanthroline moiety (by degrees). It can also be seen from the figure that two anions are on one side of the BTPhen ligand and the third is on the opposite side of these. This can explain the tilting of the two triazine groups as the two anions at the same side pull the metal cation more towards themselves.

**Figure 3:**
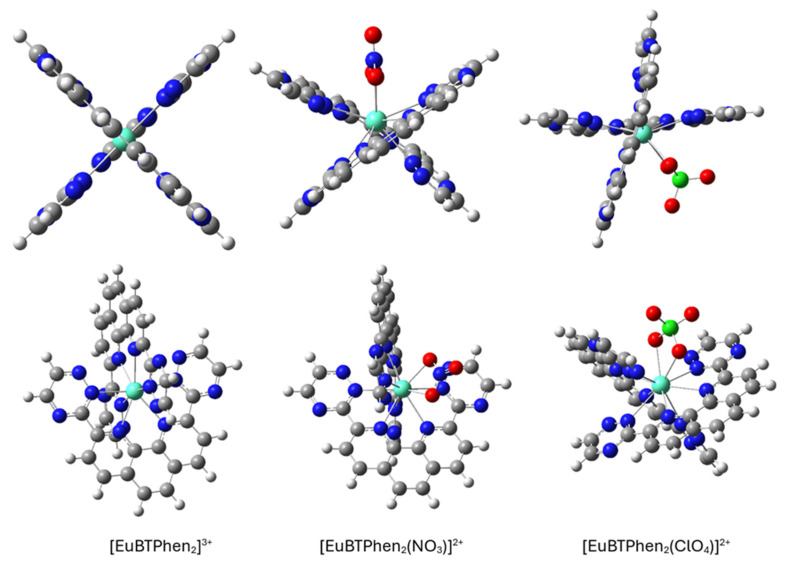
Optimized structures of the europium 2:1 complexes at the PBE1PBE/cc-pVDZ/RECP level of theory. The structures are presented from two different perspectives.

The 2:1 complex formation has been proposed by various titration and extraction studies [7,30] and determined in the solid state by X-ray diffraction [28]. This type of complex occurs when the ligand molecules are in excess compared to metal cations. Here, we have studied three different 2:1 complexes, namely the pure 2:1 BTPhen–metal cation complex, and the ones containing extra nitrate or perchlorate ions as described experimentally by X-ray diffraction [30] for nitrate ions. The optimized complex structures can be seen in Figure 3.

In [M(BTPhen)_2_]^3+^ the two ligands are perpendicular to each other and planar, while in [M(BTPhen)_2_(NO_3_)]^2+^ and [M(BTPhen)_2_(ClO_4_)]^2+^ the ligand molecules have twisted to accommodate the nitrate anion. Also, the metal cation is no longer directly in the center between two ligand planes but looks torn out towards the anion. The ligands themselves have been somewhat distorted (slightly bent away) from planarity as indicated in Figure 3. The coordination number increases from 8 to 10 with the addition of the anions.

The energies and geometries of all studied species are given in the Supplementary Information.

In terms of absolute energies, corresponding to reactions in Table 1 among the 1:1 (ratio of the metal cation–BTPhen ligand) complexes, the weakest binding corresponds to M^3+^-BTPhen complexes and the stronger ones to the complexes, where three anions (nitrates or perchlorates) are present. Such behavior is to be expected, as those complexes are electroneutral, while the M^3+^-BTPhen and M^3+^-BTPhen-4H_2_O complexes have a +3 charge. Bringing together of oppositely charged metal cations and nitrate or perchlorate anions should be energetically beneficial alongside the saturation of the remaining coordination sites. Comparing the two anions (still in 1:1 M:BTPhen complexes), one can see that for both metals, the complexation with nitrate is ca 60 kcal/mol preferred, but for americium the preference is somewhat (ca 1 kcal/mol) weaker.

For the 1:2 (metal cation: BTPhen ligand) complexes similar trends can be observed (see Table 2), although for complexes without anions the complexation energies refer to stronger binding (there are more bonds between M and BTPhen ligands) compared to 1:1 complexes and for anion containing complexes the binding is stronger than without anions (again, the bringing together of opposite charges should be beneficial alongside the saturation of the remaining coordination sites), but the differences are smaller as there are fewer anions (one versus three) involved. Interestingly enough, the differences between nitrate and perchlorate containing complexes are close to three times weaker (ca 20 kcal/mol vs. 60 kcal/mol for 1:2 vs. 1:1 complexes, respectively) in analogy with the number of anions involved. It suggests that the stabilizing ability of anions should be additive in contrast to the BTPhen, where the second ligand has a much smaller stabilizing effect (ca 40% of that of the first ligand).

The complexation strengths of Eu^3+^ and Am^3+^ can be compared by the use of metal cation exchange reactions (see Table 3), which give directly the energetic difference for metal binding. The binding of Eu^3+^ with all studied combinations of ligands is stronger (the energies of reactions Table 3 are positive) than that of Am^3+^ by 8.16 kcal/mol in the gas phase. The biggest differences correspond to the binding of metal cation with one or two BTPhen ligands (the weakest complexes), while the additional secondary ligands (water, nitrates, or perchlorates) diminish the difference, especially when the secondary ligand is anionic (and therefore the complexation energy more negative). Usually, the stronger binding means smaller difference, with the exception for comparison of nitrate and perchlorate. Interestingly enough, the perchlorate ligand brings the complexation energies of two metal complexes somewhat closer (diminishes the preferential binding of Eu^3+^). Therefore, for MBTPhen_(1-2)_X_(3,1)_ complexes (i.e., the complexes where metal cation is bound to one or two BTPhen ligands and three or one anions, respectively) the perchlorate ion(s) in complex gives extra 0.6 to 0.9 kcal/mol preference for americium, in contrast with the experimental findings, where only the nitrate solutions give preferable extraction of americium cations [29].

A more realistic model system for comparison with the solution experiments would be complex formation, where the metal cation is not a naked cation but rather bound to three nitrate anions so that it is in a neutral form, and perhaps also hydrated with at least some water molecules [26]. However, one should keep in mind that if we make such a change, it will shift the metal cation exchange energies in Table 3 just by a constant increment, making them more negative. The same applies to the differences between the complexation of different ligands with europium and americium cations. The increment directly corresponds to the reactions in Table 4.

Comparison of the binding of the two anions shows that in the case of naked salts (no water molecules included) the preferences for europium over americium are similar (14.7–14.8 kcal/mol in ΔH scale). However, the addition of four explicit water molecules creates a difference—in the case of nitrates, the preference for europium somewhat increases while for perchlorates there is a 2.6 kcal/mol decrease in the preference for the same cation.

As the binding of Eu^3+^ with the studied anions (with or without the inclusion of four water molecules, see Table 4) is considerably stronger than that of Am^3+^ (the cation exchange energies in Table 4 favor Eu(III) binding with anions and water molecules over Am(III) by 12–15 kcal/mol), it also follows that the energies of the metal exchange reactions of BTPhen complexes should be shifted in the same direction (i.e., complexation of BTPhen with americium becomes more favorable compared to europium); one should expect that the preference of BTPhen ligands to bind with europium cation should be diminished or even reversed in favor of americium. Indeed, for most complexes (exceptions are only hypothetical coordinatively unsaturated [MBTPhen]^3+^ complexes) after considering the metal nitrates, especially hydrated ones, the binding with americium might become preferred, thus also offering confirmation that americium should be the preferred complex forming cation (both for nitrates and perchlorates).

Indeed, as can be seen from the reaction energies in Table 5, in all cases (the only exceptions are two coordinatively unsaturated [MBTPhen]^3+^ complexes, which are thus not really relevant to the solution situation) the americium is preferably complexed with BTPhen, due to the stronger complexation of the europium cation with nitrate or perchlorate anions (with or without explicit water molecules present).

The preference of BTPhen binding Am(III) over Eu(III) is, however, small (less than 7 kcal/mol in both enthalpy and Gibbs free energy scale, see Table 5) and therefore even the small reaction energy changes, caused by the substitution of nitrate counterions by perchlorate, should diminish that preference and lessen the separation factor in agreement with the experiment. Indeed, that is what we see—the nitrate gives a bigger preference of americium complexation by BTPhen by 2.5 kcal/mol for enthalpy and by 1.2 kcal/mol for Gibbs free energy scale compared to perchlorate. That leads to the conclusion that the preferential extraction of Am(III) over Eu(III) stems from the delicate balance of the complexation energies of those cations with BTPhen, either in 1:1 or 1:2 complexes, and the interaction of “free” ions with counterions and water molecules.

## 3. Materials and Methods

All calculations were performed using the Gaussian 16 program package [31]. Stuttgart/Cologne energy-consistent quasi-relativistic small core pseudopotentials and corresponding basis sets [32] were used. ECP28MWB pseudopotential [33] replacing 28 core electrons and ECP28MWB_SEG (14s13p10d8f6g)/[10s9p5d4f3g] basis set [34] was used for europium, while ECP60MWB pseudopotential [35] replacing 60 core electrons and ECP60MWB_SEG (14s13p10d8f6g)/[10s9p5d4f3g] basis set [36,37] was used for americium. Cc-pVDZ basis set [38] was used for other atoms. All structures were optimized and frequency calculations were performed to ensure that the obtained structures correspond to minima (number of imaginary frequencies equals zero). All possible conformers (whenever feasible) were used as starting geometries to ensure that the global minima on the potential energy surfaces were found. The resulting frequencies were used to calculate enthalpies and Gibbs free energies. Thermodynamic calculations were carried out at 298.15 K and 1 atm of pressure. All calculations involving the europium or americium were carried out for the septet state.

## 4. Conclusions

In the current study, DFT calculations were carried out for 1:1 and 2:1 complexes of Eu^3+^/Am^3+^ and the BTPhen ligand. The energetic calculations were carried out with “the naked” M^3+^ cation as well as M(NO_3_)_3_(H_2_O)_4_ complexes and it was found that the latter better describes the complexation reaction that occurs in the solution in agreement with our earlier study [26]. In calculations with the M(NO_3_)_3_(H_2_O)_4_ complex, Am^3+^ formed the more stable complexes in all of the studied complexation forms. This stems from the fact that in M(NO_3_)_3_(H_2_O)_4_ europium gets a bigger stabilizing effect when compared to americium and thus the latter reacts more easily with the BTPhen ligand.

The comparison of counterion influence (nitrate vs. perchlorate) indicates that bigger preference for americium over europium complexation corresponds to the nitrate complexes (ca 3 kcal/mol in energy, 2.5 kcal/mol in enthalpy and 1.2 kcal/mol in Gibbs free energy scale). The preferential extraction from nitrate containing solutions over the perchlorate containing ones is therefore at least qualitatively confirmed and stems mainly from the fact that in M(NO_3_)_3_(H_2_O)_4_ the europium is stabilized more than in M(ClO_4_)_3_(H_2_O)_4_.

## Figures and Tables

**Table 1:** The energies for 1:1 complex formation (in kcal/mol).

	ΔE	ΔH	ΔG
Eu^3+^ + BTPhen = [BTPhen–Eu]^3+^	−484.5	−483.6	−471.7
Eu^3+^ + BTPhen + 3NO_3_^−^ = [BTPhen–Eu–3NO_3_]	−1117.3	−1111.6	−1066.9
Eu^3+^ + BTPhen + 3ClO_4_^−^ = [BTPhen–Eu–3ClO_4_]	−1057.6	−1052.3	−1005.2
Eu^3+^ + BTPhen + 4H_2_O = [BTPhen–Eu–4H_2_O]^3+^	−645.4	−637.0	−583.8
Am^3+^ + BTPhen = [BTPhen–Am]^3+^	−469.7	−468.1	−455.4
Am^3+^ + BTPhen + 3NO_3_^−^ = [BTPhen–Am–3NO_3_]	−1107.4	−1101.9	−1057.4
Am^3+^ + BTPhen + 3ClO_4_^−^ = [BTPhen–Am–3ClO_4_]	−1048.5	−1043.4	−996.7
Am^3+^ + BTPhen + 4H_2_O = [BTPhen–Am–4H_2_O]^3+^	−633.8	−625.6	−572.8

**Table 2:** The energies for 1:2 complex formation (in kcal/mol).

	ΔE	ΔH	ΔG
Eu^3+^ + 2BTPhen = [BTPhen_2_–Eu]^3+^	−672.4	−667.8	−638.8
Eu^3+^ + 2BTPhen + NO_3_^−^ = [BTPhen_2_–Eu–NO_3_]^2+^	−896.4	−890.6	−850.5
Eu^3+^ + 2BTPhen + ClO_4_^−^ = [BTPhen_2_–Eu–ClO_4_]^2+^	−876.2	−870.4	−829.3
Am^3+^ + 2BTPhen = [BTPhen_2_–Am]^3+^	−660.5	−656.0	−627.8
Am^3+^ + 2BTPhen + NO_3_^−^ = [BTPhen_2_–Am–NO_3_]^2+^	−887.6	−881.8	−841.4
Am^3+^ + 2BTPhen + ClO_4_^−^ = [BTPhen_2_–Am–ClO_4_]^2+^	−868.0	−862.3	−820.9

**Table 3:** The energies for cation exchange reactions (in kcal/mol).

	ΔE	ΔH	ΔG
Am^3+^ + [EuBTPhen]^3+^ = [AmBTPhen]^3+^ + Eu^3+^	14.8	15.5	16.3
Am^3+^ + [Eu(H_2_O)_4_BTPhen]^3+^ = [Am(H_2_O)_4_BTPhen]^3+^ + Eu^3+^	11.5	11.4	10.9
Am^3+^ + [Eu(NO_3_)_3_BTPhen] = [Am(NO_3_)_3_BTPhen] + Eu^3+^	9.9	9.7	9.4
Am^3+^ + [Eu(ClO_4_)_3_BTPhen] = [Am(ClO_4_)_3_BTPhen] + Eu^3+^	9.1	8.9	8.6
Am^3+^ + [Eu(BTPhen)_2_]^3+^ = [Am(BTPhen)_2_]^3+^ + Eu^3+^	11.9	11.8	11.0
Am^3+^ + [Eu(BTPhen)_2_(NO_3_)]^2+^ = [Am(BTPhen)_2_(NO_3_)]^2+^ + Eu^3+^	8.8	8.8	9.2
Am^3+^ + [Eu(BTPhen)_2_(ClO_4_)]^2+^ = [Am(BTPhen)_2_(ClO_4_)]^2+^ + Eu^3+^	8.2	8.2	8.4

**Table 4:** The energies for cation exchange reactions for salts (in kcal/mol).

	ΔE	ΔH	ΔG
Am^3+^ + Eu(ClO_4_)_3_ = Eu^3+^ + Am(ClO_4_)_3_	15.1	14.9	14.4
Am^3+^ + Eu(NO_3_)_3_ = Eu^3+^ + Am(NO_3_)_3_	14.8	14.7	14.8
Am^3+^ + Eu(ClO_4_)_3_·4H_2_O = Eu^3+^ + Am(ClO_4_)_3_·4H_2_O	12.5	12.3	11.8
Am^3+^ + Eu(NO_3_)_3_·4H_2_O = Eu^3+^ + Am(NO_3_)_3_·4H_2_O	15.2	14.7	13.0

**Table 5:** Comparison of cation exchange energies for anions in used salts (nitrate vs. perchlorate, in kcal/mol).

	L = NO_3^−^_			L = ClO_4^−^_		
	ΔE	ΔH	ΔG	ΔE	ΔH	ΔG
AmL_3_ + [EuBTPhen]^3+^ = [AmBTPhen]^3+^ + EuL_3_	−0.1	0.8	1.5	−0.3	0.6	1.9
AmL_3_ + [Eu(H_2_O)_4_BTPhen]^3+^ = [Am(H_2_O)_4_BTPhen]^3+^ + EuL_3_	−3.3	−3.3	−3.9	−3.5	−3.4	−3.5
AmL_3_ + [Eu(NO_3_)_3_BTPhen] = [Am(NO_3_)_3_BTPhen] + EuL_3_	−4.9	−4.9	−5.4	−5.2	−5.1	−5.0
AmL_3_ + [Eu(ClO_4_)_3_BTPhen] = [Am(ClO_4_)_3_BTPhen] + EuL_3_	−5.8	−5.8	−6.3	−6.0	−5.9	−5.9
AmL_3_ + [Eu(BTPhen)_2_]^3+^ = [Am(BTPhen)_2_]^3+^ + EuL_3_	−3.0	−2.9	−3.8	−3.2	−3.1	−3.5
AmL_3_ + [Eu(BTPhen)_2_(NO_3_)]^2+^ = [Am(BTPhen)_2_(NO_3_)]^2+^ + EuL_3_	−6.0	−5.9	−5.6	−6.2	−6.1	−5.3
AmL_3_ + [Eu(BTPhen)_2_(ClO_4_)]^2+^ = [Am(BTPhen)_2_(ClO_4_)]^2+^ + EuL_3_	−6.6	−6.5	−6.4	−6.8	−6.7	−6.0
AmL_3_·4H_2_O + [EuBTPhen]^3+^ = [AmBTPhen]^3+^ + EuL_3_·4H_2_O	−0.4	0.8	3.3	2.3	3.2	4.6
AmL_3_·4H_2_O + [Eu(H_2_O)_4_BTPhen]^3+^ = [Am(H_2_O)_4_BTPhen]^3+^ + EuL_3_·4H_2_O	−3.7	−3.3	−2.1	−0.9	−0.8	−0.8
AmL_3_·4H_2_O + [Eu(NO_3_)_3_BTPhen] = [Am(NO_3_)_3_BTPhen] + EuL_3_·4H_2_O	−5.3	−5.0	−3.6	−2.6	−2.5	−2.3
AmL_3_·4H_2_O + [Eu(ClO_4_)_3_BTPhen] = [Am(ClO_4_)_3_BTPhen] + EuL_3_·4H_2_O	−6.1	−5.8	−4.5	−3.4	−3.3	−3.2
AmL_3_·4H_2_O + [Eu(BTPhen)_2_]^3+^ = [Am(BTPhen)_2_]^3+^ + EuL_3_·4H_2_O	−3.3	−2.9	−2.0	−0.6	−0.5	−0.8
AmL_3_·4H_2_O + [Eu(BTPhen)_2_(NO_3_)]^2+^ = [Am(BTPhen)_2_(NO_3_)]^2+^ + EuL_3_·4H_2_O	−6.4	−5.9	−3.8	−3.6	−3.5	−2.6
AmL_3_·4H_2_O + [Eu(BTPhen)_2_(ClO_4_)]^2+^ = [Am(BTPhen)_2_(ClO_4_)]^2+^ + EuL_3_·4H_2_O	−7.0	−6.6	−4.6	−4.3	−4.1	−3.4

## Data Availability

The original contributions presented in this study are included in the article/Supplementary Material. Further inquiries can be directed to the corresponding author.

## Supplementary Materials

The following supporting information can be downloaded at https://www.mdpi.com/article/10.3390/molecules31040665/s1, Table S1: The raw calculated energies for all studied species; Table S2: Geometries for all studied species.

## Abbreviations

The following abbreviations are used in this manuscript:

M	Metal, either europium or americium
BTPhen	2,9-bis(1,2,4-triazin-3-yl)-1,10-phenanthroline
