# Supplementary material for: DFT Study of Americium and Europium Complexation with 2,9-Bis(1,2,4-triazin-3-yl)-1,10-Phenanthroline Ligand: The Influence of the Counteranions–Nitrate Versus Perchlorate
Source: Molecules. 2026 Feb 14;31(4):665. doi: 10.3390/molecules31040665 (PMC12943513; doi:10.3390/molecules31040665)
Supplement: Supplementary file 1 [file molecules-31-00665-s001.zip › molecules-4129594-supplementary.pdf]

Table S1. Raw energies (E), enthalpies (H), and Gibbs fee energies (G) of all studied species (all in a.u.)

	E	H	G
Eu^{3+}	-708.784	-708.782	-708.803
Am^{3+}	-593.882	-593.88	-593.902
BTPhen	-1128.68	-1128.4	-1128.47
H_2O	-76.339	-76.3137	-76.3352
NO_3^-	-280.075	-280.056	-280.084
ClO_{4-}	-760.36	-760.34	-760.371
1:1 complexes			
$[EuBTPhen]^{3+}$	-1838.24	-1837.95	-1838.03
$[EuBTPhen\text{-}3NO_3]$	-2679.47	-2679.12	-2679.23
$[EuBTPhen\text{-}4H_2O]^{3+}$	-2143.85	-2143.45	-2143.54
$[EuBTPhen\text{-}3ClO_4]$	-4120.23	-4119.88	-4119.99
$[AmBTPhen]^{3+}$	-1723.31	-1723.03	-1723.1
$[AmBTPhen\text{-}3NO_3]$	-2564.55	-2564.21	-2564.31
$[AmBTPhen\text{-}4H_2O]^{3+}$	-2028.93	-2028.53	-2028.63
$[AmBTPhen\text{-}3ClO_4]$	-4005.31	-4004.96	-4005.07
2:1 complexes			
$[EuBTPhen_2]^{3+}$	-2967.22	-2966.65	-2966.76
$[EuBTPhen_2\text{-}NO_3]^{2+}$	-3247.65	-3247.06	-3247.18
$[EuTPhen_2\text{-}ClO_4]^{2+}$	-3727.9	-3727.31	-3727.44
$[AmBTPhen_2]^{3+}$	-2852.3	-2851.73	-2851.84
$[AmBTPhen_2\text{-}NO_3]^{2+}$	-3132.73	-3132.14	-3132.27
$[AmBTPhen_2\text{-}ClO_4]^{2+}$	-3612.99	-3612.4	-3612.52
Salts			
$Eu(ClO_4)_3$	-2991.44	-2991.37	-2991.44
$Eu(NO_3)_3$	-1550.69	-1550.63	-1550.69
$Am(ClO_4)_3$	-2876.51	-2876.45	-2876.52
$Am(NO_3)_3$	-1435.76	-1435.7	-1435.76
$Eu(ClO_4)_3(H_2O)_4$	-3296.96	-3296.78	-3296.87
$Eu(NO_3)_3(H_2O)_4$	-1856.2	-1856.02	-1856.1
$Am(ClO_4)_3(H_2O)_4$	-3182.04	-3181.86	-3181.95
$Am(NO_3)_3(H_2O)_4$	-1741.27	-1741.09	-1741.18

Table S2. Geometries of all studied species (all in Å).

	$[EuBTPhen]^{3+}$				$[AmBTPhen]^{3+}$		
Eu	0.00000	1.26168	0.00000	Am	0.0000	0.0000	1.1039
N	-2.80594	2.75028	-0.00001	N	0.0000	-2.6671	2.5920
N	-2.42121	1.50765	0.00000	N	0.0000	-2.3538	1.3148
N	-1.35011	-0.85823	0.00000	N	0.0000	-1.3418	-1.0164
N	1.35011	-0.85823	0.00000	N	0.0000	1.3418	-1.0164
N	2.42121	1.50765	0.00000	N	0.0000	2.3538	1.3148
N	2.80594	2.75028	0.00001	N	0.0000	2.6671	2.5920
C	-5.02550	1.94615	0.00002	C	0.0000	-4.9267	1.8639
C	-4.09962	3.02270	0.00000	C	0.0000	-3.9533	2.8992
C	-3.29879	0.48924	-0.00001	C	0.0000	-3.2744	0.3446
C	-2.69479	-0.85034	0.00000	C	0.0000	-2.6906	-1.0045
C	-3.47316	-2.01327	0.00000	C	0.0000	-3.4699	-2.1645
H	-4.56165	-1.93428	0.00000	H	0.0000	-4.5581	-2.0803
C	-2.82796	-3.23901	0.00000	C	0.0000	-2.8286	-3.3939
H	-3.40673	-4.16586	-0.00001	H	0.0000	-3.4113	-4.3184
C	-1.41899	-3.28888	-0.00001	C	0.0000	-1.4196	-3.4490
C	-0.72021	-2.05344	0.00000	C	0.0000	-0.7179	-2.2166
C	-0.68130	-4.51874	0.00000	C	0.0000	-0.6815	-4.6790
H	-1.23307	-5.46089	0.00001	H	0.0000	-1.2330	-5.6213
C	0.68130	-4.51874	0.00000	C	0.0000	0.6815	-4.6790
H	1.23307	-5.46089	-0.00001	H	0.0000	1.2330	-5.6213
C	0.72021	-2.05344	0.00000	C	0.0000	0.7179	-2.2166
C	1.41899	-3.28888	0.00001	C	0.0000	1.4196	-3.4490
C	2.82796	-3.23901	0.00000	C	0.0000	2.8286	-3.3939
H	3.40673	-4.16586	0.00001	H	0.0000	3.4113	-4.3184
C	3.47316	-2.01327	0.00000	C	0.0000	3.4699	-2.1645
H	4.56165	-1.93428	0.00000	H	0.0000	4.5581	-2.0803
C	2.69479	-0.85034	0.00000	C	0.0000	2.6906	-1.0045
C	3.29879	0.48924	0.00001	C	0.0000	3.2744	0.3446
C	4.09962	3.02270	0.00000	C	0.0000	3.9533	2.8992
C	5.02550	1.94615	-0.00002	C	0.0000	4.9267	1.8639
H	-6.10604	2.13237	0.00001	H	0.0000	-5.9962	2.1012
H	-4.40115	4.07294	0.00000	H	0.0000	-4.2166	3.9597
H	4.40115	4.07294	0.00000	H	0.0000	4.2166	3.9597
H	6.10604	2.13237	-0.00001	H	0.0000	5.9962	2.1012
N	-4.61255	0.69263	-0.00001	N	0.0000	-4.5831	0.5844
N	4.61255	0.69263	0.00001	N	0.0000	4.5831	0.5844
	$[EuBTPhen-3NO_3]$				$[AmBTPhen-3NO_3]$		
Eu	0.11308	-0.83786	0.12893	Am	-0.1396	-0.7631	0.1007
H	6.45371	-0.49406	-0.44776	H	-6.4676	-0.1715	-0.4322
H	5.18762	-2.71990	-0.51402	H	-5.2944	-2.4468	-0.5265
H	-6.03962	-1.92326	-0.92189	H	6.0066	-2.0385	-0.8948
H	-4.29613	-3.77429	-1.24154	H	4.2099	-3.8356	-1.2267
H	-4.74912	1.94630	-0.37470	H	4.8344	1.8701	-0.3652
H	-3.86643	4.29227	-0.12109	H	4.0254	4.2407	-0.0998
H	0.57115	6.10539	0.10944	H	-0.3497	6.1944	0.1397
H	-1.88328	5.81119	0.05179	H	2.0944	5.8209	0.0808
H	2.86155	5.10050	0.04155	H	-2.6679	5.2695	0.0661
H	4.28636	3.02680	-0.10330	H	-4.1644	3.2485	-0.0913
C	0.47184	2.66806	-0.08686	C	-0.3634	2.7565	-0.0793
C	1.00853	3.97817	-0.01721	C	-0.8555	4.0837	-0.0008

C	0.13194	5.10811	0.04227	C	0.0573	5.1840	0.0659
C	-1.21824	4.94631	0.01040	C	1.4013	4.9787	0.0334
C	-1.79953	3.64210	-0.08907	C	1.9389	3.6568	-0.0746
C	-0.96602	2.49655	-0.12751	C	1.0687	2.5392	-0.1217
C	2.56526	1.72516	-0.15210	C	-2.4915	1.8872	-0.1489
C	3.19847	2.98140	-0.09146	C	-3.0789	3.1648	-0.0804
C	2.41152	4.10712	-0.01527	C	-2.2529	4.2614	0.0027
C	-2.77153	1.08614	-0.33827	C	2.8303	1.0724	-0.3397
C	-3.68394	2.15766	-0.29654	C	3.7761	2.1143	-0.2888
C	-3.19107	3.43500	-0.16099	C	3.3233	3.4055	-0.1462
C	3.37357	0.48814	-0.23953	C	-3.3485	0.6837	-0.2433
C	4.67734	-1.75883	-0.42887	C	-4.7446	-1.5081	-0.4396
C	5.36158	-0.54019	-0.39735	C	-5.3780	-0.2625	-0.3918
C	-3.26375	-0.29520	-0.54924	C	3.2796	-0.3232	-0.5542
C	-4.02088	-2.73970	-1.02900	C	3.9648	-2.7920	-1.0221
C	-4.96510	-1.72522	-0.86278	C	4.9384	-1.8067	-0.8473
N	1.24832	1.57329	-0.14022	N	-1.1793	1.6892	-0.1378
N	-1.45874	1.25021	-0.23368	N	1.5233	1.2791	-0.2370
N	2.70204	-0.66884	-0.24931	N	-2.7252	-0.5001	-0.2724
N	3.35396	-1.80598	-0.34728	N	-3.4236	-1.6094	-0.3731
N	-2.34276	-1.26035	-0.67645	N	2.3301	-1.2584	-0.6974
N	-2.71954	-2.49195	-0.93204	N	2.6710	-2.5023	-0.9451
N	0.45880	-1.27164	-2.69690	N	0.9735	0.1672	2.6602
N	-0.92097	0.15434	2.64929	N	-0.4631	-1.1535	-2.7782
N	0.59192	-3.18282	1.64725	N	-0.7405	-3.1165	1.6511
O	0.54147	-2.20144	-1.83975	O	-0.0840	0.6557	2.1488
O	0.20140	-0.11718	-2.22760	O	1.5442	-0.7361	1.9745
O	0.61372	-1.46200	-3.87467	O	1.4105	0.5512	3.7118
O	-1.53877	-0.71693	1.96303	O	-0.2018	-0.0072	-2.2870
O	0.16004	0.58559	2.14168	O	-0.5671	-2.0940	-1.9333
O	-1.33947	0.55823	3.70193	O	-0.6013	-1.3263	-3.9597
O	1.27229	-2.11478	1.80189	O	-1.3355	-2.0045	1.8434
O	-0.30663	-3.12724	0.75572	O	0.1231	-3.1132	0.7196
O	0.79528	-4.16728	2.30191	O	-0.9834	-4.0927	2.3036
N	4.70527	0.60429	-0.30416	N	4.5949	-0.5485	-0.6226
N	-4.58468	-0.47852	-0.63335	N	-4.6748	0.8535	-0.2947
	**[EuBTPhen-4H_2O]$^{3+}$**				**[AmBTPhen-4H_2O]$^{3+}$**		
H	6.29577	1.56369	0.07775	H	6.3428	1.3762	0.0794
H	4.79702	3.57059	-0.52182	H	4.8718	3.4227	-0.4568
H	-6.29578	1.56367	-0.07777	H	-6.3428	1.3762	-0.0794
H	-4.79704	3.57057	0.52179	H	-4.8718	3.4227	0.4568
H	-4.54434	-2.21046	-0.35396	H	-4.5505	-2.3505	-0.3041
H	-3.38373	-4.43857	-0.33162	H	-3.3799	-4.5738	-0.2804
H	1.22827	-5.72679	0.12187	H	1.2308	-5.8505	0.1044
H	-1.22825	-5.72679	-0.12186	H	-1.2308	-5.8505	-0.1044
H	3.38375	-4.43856	0.33162	H	3.3799	-4.5738	0.2804
H	4.54436	-2.21044	0.35395	H	4.5505	-2.3505	0.3040
C	0.71862	-2.31851	0.06003	C	0.7202	-2.4396	0.0464
C	1.40976	-3.55554	0.13197	C	1.4092	-3.6795	0.1097
C	0.67756	-4.78578	0.06710	C	0.6780	-4.9103	0.0571
C	-0.67754	-4.78578	-0.06709	C	-0.6780	-4.9103	-0.0571
C	-1.40975	-3.55555	-0.13197	C	-1.4092	-3.6795	-0.1097
C	-0.71861	-2.31852	-0.06003	C	-0.7202	-2.4396	-0.0464
C	2.69142	-1.11978	0.17140	C	2.7009	-1.2523	0.1375

C	3.45955	-2.28879	0.27721	C	3.4651	-2.4245	0.2342
C	2.81296	-3.50985	0.25990	C	2.8135	-3.6418	0.2183
C	-2.69141	-1.11979	-0.17140	C	-2.7009	-1.2523	-0.1375
C	-3.45953	-2.28880	-0.27721	C	-3.4651	-2.4245	-0.2342
C	-2.81294	-3.50986	-0.25990	C	-2.8135	-3.6418	-0.2183
C	3.34575	0.20087	0.07442	C	3.3695	0.0619	0.0601
C	4.39191	2.57657	-0.31928	C	4.4529	2.4300	-0.2772
C	5.20883	1.46642	-0.00933	C	5.2539	1.2992	-0.0030
C	-3.34575	0.20086	-0.07443	C	-3.3695	0.0619	-0.0602
C	-4.39192	2.57655	0.31927	C	-4.4529	2.4300	0.2772
C	-5.20883	1.46640	0.00931	C	-5.2539	1.2992	0.0029
N	1.35433	-1.12486	0.08673	N	1.3625	-1.2483	0.0592
N	-1.35432	-1.12487	-0.08673	N	-1.3625	-1.2483	-0.0592
N	2.54441	1.24688	-0.15510	N	2.5834	1.1261	-0.1322
N	3.07950	2.43933	-0.37867	N	3.1388	2.3145	-0.3307
N	-2.54442	1.24687	0.15510	N	-2.5834	1.1261	0.1322
N	-3.07951	2.43932	0.37866	N	-3.1388	2.3145	0.3307
Eu	-0.00001	0.99587	0.00000	Am	0.0000	0.9273	0.0000
O	0.23322	0.56989	2.36204	O	0.3176	0.5428	2.4227
O	-0.23318	0.56989	-2.36203	O	-0.3176	0.5428	-2.4227
O	-0.65011	2.93965	1.34351	O	-0.7562	2.8788	1.3037
O	0.65007	2.93969	-1.34346	O	0.7562	2.8788	-1.3036
H	-0.49233	-0.19688	-2.89257	H	-0.6679	-0.1911	-2.9486
H	0.08940	1.23805	-2.98679	H	0.0270	1.1847	-3.0635
H	-0.21933	3.77269	1.58201	H	-0.3480	3.7188	1.5582
H	-1.59602	3.15649	1.13615	H	-1.7047	3.0712	1.0751
H	0.21930	3.77273	-1.58193	H	0.3480	3.7189	-1.5581
H	1.59599	3.15652	-1.13610	H	1.7047	3.0712	-1.0751
H	0.49242	-0.19688	2.89255	H	0.6679	-0.1912	2.9486
H	-0.08938	1.23802	2.98681	H	-0.0270	1.1846	3.0635
N	-4.67796	0.27175	-0.16718	N	-4.7041	0.1097	-0.1432
N	4.67796	0.27177	0.16716	N	4.7041	0.1098	0.1431
	**[EuBTPhen-3ClO$_4$]**				**[AmBTPhen-3ClO$_4$]**		
Eu	0.45700	-0.40954	-0.07084	Am	0.4453	-0.3589	-0.0558
H	5.14062	3.87242	0.01059	H	4.9517	4.1209	-0.0882
H	5.57397	1.35041	0.15251	H	5.4962	1.6231	0.0929
H	-3.32721	-5.01417	2.05761	H	-3.2244	-5.0865	2.0753
H	-0.77681	-5.25616	2.13631	H	-0.6681	-5.2536	2.1645
H	-4.95270	-1.35956	0.99872	H	-4.9523	-1.4870	1.0222
H	-5.82823	0.94928	0.50267	H	-5.9031	0.7880	0.5109
H	-3.64726	5.15386	-0.31175	H	-3.8686	5.0520	-0.3560
H	-5.31786	3.36318	0.02170	H	-5.4768	3.2094	0.0053
H	-1.23817	5.86453	-0.42767	H	-1.4930	5.8432	-0.4939
H	1.19240	5.20242	-0.33942	H	0.9605	5.2718	-0.4131
C	-1.49088	2.49222	0.01462	C	-1.6203	2.4701	-0.0012
C	-1.92918	3.82850	-0.15161	C	-2.1068	3.7877	-0.1848
C	-3.33076	4.12014	-0.15974	C	-3.5172	4.0316	-0.1915
C	-4.25042	3.13426	0.02484	C	-4.4021	3.0177	0.0082
C	-3.84325	1.77987	0.24632	C	-3.9473	1.6813	0.2440
C	-2.46738	1.44887	0.23140	C	-2.5609	1.3969	0.2311
C	0.71910	3.11146	-0.09985	C	0.5667	3.1697	-0.1389
C	0.38989	4.47132	-0.25326	C	0.1859	4.5134	-0.3102
C	-0.94024	4.82238	-0.29473	C	-1.1561	4.8148	-0.3478
C	-2.90067	-0.74958	0.74820	C	-2.9214	-0.8121	0.7662

C	-4.28940	-0.52732	0.76922	C	-4.3166	-0.6351	0.7867
C	-4.75679	0.73886	0.50136	C	-4.8253	0.6129	0.5100
C	2.13173	2.67904	-0.04700	C	1.9970	2.7979	-0.0933
C	4.56842	1.76963	0.08979	C	4.4728	1.9969	0.0331
C	4.32373	3.14455	0.01458	C	4.1673	3.3585	-0.0630
C	-2.35176	-2.07719	1.10195	C	-2.3321	-2.1214	1.1247
C	-1.26915	-4.33285	1.82621	C	-1.1862	-4.3450	1.8531
C	-2.65779	-4.19081	1.79099	C	-2.5784	-4.2436	1.8120
N	-0.19079	2.15373	0.01306	N	-0.3071	2.1797	-0.0047
N	-2.02164	0.20210	0.46099	N	-2.0748	0.1671	0.4721
N	2.34730	1.36094	0.01111	N	2.2710	1.4915	-0.0155
N	3.57301	0.89470	0.07604	N	3.5165	1.0794	0.0457
N	-1.01733	-2.20552	1.09640	N	-0.9945	-2.2105	1.1254
N	-0.46668	-3.33645	1.47142	N	-0.4123	-3.3250	1.5027
O	-0.59182	0.60566	-2.12387	O	-0.6479	0.5831	-2.1510
O	-1.02917	-1.68851	-1.63967	O	-1.0349	-1.7220	-1.6509
O	-2.83556	-0.28945	-2.53091	O	-2.8740	-0.3639	-2.5405
O	0.18444	0.52506	2.25300	O	0.1266	0.5871	2.2997
O	1.91681	-1.04213	1.80893	O	1.9467	-0.8951	1.8827
O	2.40453	0.90784	3.22594	O	2.3267	1.1108	3.2543
O	1.98163	-0.70598	-1.93461	O	2.0062	-0.6853	-1.9536
O	1.57070	-2.54617	-0.48526	O	1.6376	-2.4915	-0.4428
O	3.81706	-2.26982	-1.45243	O	3.8799	-2.1872	-1.4146
Cl	-1.39690	-0.58944	-2.63785	Cl	-1.4302	-0.6365	-2.6527
O	-0.99532	-0.95181	-3.99234	O	-1.0279	-0.9948	-4.0082
Cl	2.38355	-2.17702	-1.72891	Cl	2.4458	-2.1378	-1.6993
Cl	1.40334	-0.12222	2.91306	Cl	1.3834	0.0198	2.9678
O	0.99839	-0.89696	4.08686	O	1.0245	-0.7487	4.1604
O	1.96911	-2.98159	-2.87516	O	2.0558	-2.9927	-2.8175
N	-3.21215	-3.04297	1.43507	N	2.9124	3.7712	-0.1313
N	3.08868	3.61251	-0.05968	N	-3.1648	-3.1125	1.4544
	**$[EuBTPhen_2]^{3+}$**				**$[AmBTPhen_2]^{3+}$**		
Eu	0.00000	0.00000	0.00000	Am	0.0000	0.0000	0.0000
H	-4.44772	4.42872	-0.58378	H	0.5029	4.4860	4.4316
H	-3.32613	3.37722	-2.63758	H	2.5698	3.3571	3.4146
H	4.44772	-4.42872	-0.58378	H	0.5029	-4.4860	-4.4316
H	3.32613	-3.37722	-2.63758	H	2.5698	-3.3571	-3.4146
H	3.24747	-3.19765	3.21350	H	-3.2698	-3.2663	-3.1821
H	2.42610	-2.37684	5.44335	H	-5.4955	-2.4366	-2.3581
H	-0.88162	0.86416	6.72838	H	-6.7726	0.8870	0.8594
H	0.88162	-0.86416	6.72838	H	-6.7726	-0.8870	-0.8594
H	-2.42610	2.37684	5.44335	H	-5.4955	2.4366	2.3581
H	-3.24747	3.19765	3.21350	H	-3.2698	3.2663	3.1821
C	-0.51605	0.50401	3.32126	C	-3.3637	0.5204	0.5004
C	-1.01114	0.99018	4.55749	C	-4.6020	1.0163	0.9828
C	-0.48612	0.47626	5.78784	C	-5.8326	0.4887	0.4732
C	0.48612	-0.47626	5.78784	C	-5.8326	-0.4887	-0.4732
C	1.01114	-0.99018	4.55749	C	-4.6020	-1.0163	-0.9828
C	0.51605	-0.50401	3.32126	C	-3.3637	-0.5204	-0.5004
C	-1.92097	1.89065	2.12390	C	-2.1741	1.9370	1.8835
C	-2.47479	2.43329	3.29377	C	-3.3464	2.4904	2.4207
C	-2.01653	1.97685	4.51322	C	-4.5631	2.0275	1.9632
C	1.92097	-1.89065	2.12390	C	-2.1741	-1.9370	-1.8835
C	2.47479	-2.43329	3.29377	C	-3.3464	-2.4904	-2.4207

C	2.01653	-1.97685	4.51322	C	-4.5631	-2.0275	-1.9632
C	-2.36940	2.35458	0.79412	C	-0.8498	2.3908	2.3578
C	-3.05870	3.08969	-1.61869	C	1.5547	3.0868	3.1169
C	-3.67099	3.66355	-0.48889	C	0.4173	3.7032	3.6716
C	2.36940	-2.35458	0.79412	C	-0.8498	-2.3908	-2.3578
C	3.05870	-3.08969	-1.61869	C	1.5547	-3.0868	-3.1169
C	3.67099	-3.66355	-0.48889	C	0.4173	-3.7032	-3.6716
N	-0.97240	0.94962	2.13196	N	-2.1760	0.9852	0.9446
N	0.97240	-0.94962	2.13196	N	-2.1760	-0.9852	-0.9446
N	-1.77356	1.79618	-0.26886	N	0.2201	1.7903	1.8190
N	-2.12019	2.16457	-1.49217	N	1.4392	2.1407	2.1983
N	1.77356	-1.79618	-0.26886	N	0.2201	-1.7903	-1.8190
N	2.12019	-2.16457	-1.49217	N	1.4392	-2.1407	-2.1983
H	4.44771	4.42873	0.58378	H	-0.5029	-4.4860	4.4316
H	3.32610	3.37725	2.63758	H	-2.5698	-3.3571	3.4146
H	-4.44771	-4.42873	0.58378	H	-0.5029	4.4860	-4.4316
H	-3.32610	-3.37725	2.63758	H	-2.5698	3.3571	-3.4146
H	-3.24747	-3.19765	-3.21350	H	3.2698	3.2663	-3.1821
H	-2.42610	-2.37684	-5.44335	H	5.4955	2.4366	-2.3581
H	0.88163	0.86416	-6.72837	H	6.7726	-0.8870	0.8594
H	-0.88163	-0.86416	-6.72837	H	6.7726	0.8870	-0.8594
H	2.42610	2.37684	-5.44335	H	5.4955	-2.4366	2.3581
H	3.24747	3.19765	-3.21350	H	3.2698	-3.2663	3.1821
C	0.51605	0.50401	-3.32126	C	3.3637	-0.5204	0.5004
C	1.01114	0.99018	-4.55749	C	4.6020	-1.0163	0.9828
C	0.48612	0.47626	-5.78784	C	5.8326	-0.4887	0.4732
C	-0.48612	-0.47626	-5.78784	C	5.8326	0.4887	-0.4732
C	-1.01114	-0.99018	-4.55749	C	4.6020	1.0163	-0.9828
C	-0.51605	-0.50401	-3.32126	C	3.3637	0.5204	-0.5004
C	1.92096	1.89065	-2.12390	C	2.1741	-1.9370	1.8835
C	2.47479	2.43329	-3.29377	C	3.3464	-2.4904	2.4207
C	2.01653	1.97685	-4.51322	C	4.5631	-2.0275	1.9632
C	-1.92096	-1.89065	-2.12390	C	2.1741	1.9370	-1.8835
C	-2.47479	-2.43329	-3.29377	C	3.3464	2.4904	-2.4207
C	-2.01653	-1.97685	-4.51322	C	4.5631	2.0275	-1.9632
C	2.36939	2.35459	-0.79412	C	0.8498	-2.3908	2.3578
C	3.05868	3.08972	1.61869	C	-1.5547	-3.0868	3.1169
C	3.67098	3.66356	0.48890	C	-0.4173	-3.7032	3.6716
C	-2.36939	-2.35459	-0.79412	C	0.8498	2.3908	-2.3578
C	-3.05868	-3.08972	1.61869	C	-1.5547	3.0868	-3.1169
C	-3.67098	-3.66356	0.48890	C	-0.4173	3.7032	-3.6716
N	0.97240	0.94962	-2.13196	N	2.1760	-0.9852	0.9446
N	-0.97240	-0.94962	-2.13196	N	2.1760	0.9852	-0.9446
N	1.77354	1.79620	0.26886	N	-0.2201	-1.7903	1.8190
N	2.12017	2.16460	1.49217	N	-1.4392	-2.1407	2.1983
N	-1.77354	-1.79620	0.26886	N	-0.2201	1.7903	-1.8190
N	-2.12017	-2.16460	1.49217	N	-1.4392	2.1407	-2.1983
N	-3.32124	3.28979	0.72915	N	-0.7959	3.3508	3.2856
N	3.32124	-3.28979	0.72915	N	-0.7959	-3.3508	-3.2856
N	-3.32124	-3.28979	-0.72915	N	0.7959	3.3508	-3.2856
N	3.32124	3.28979	-0.72915	N	0.7959	-3.3508	3.2856
	**$[EuBTPhen_2\text{-}NO_3]^{2+}$**				**$[AmBTPhen_2\text{-}NO_3]^{2+}$**		
Eu	0.00001	-0.00003	-0.25351	Am	0.0000	0.0002	-0.2266
O	-1.04238	-0.24670	-2.43626	O	1.0558	0.1875	-2.4626

O	1.04207	0.24597	-2.43646	O	-1.0547	-0.1868	-2.4634
O	-0.00023	-0.00080	-4.32101	O	0.0017	-0.0022	-4.3486
N	0.90783	3.14168	-1.33705	N	-0.8926	-3.1350	-1.3653
N	-0.20071	2.46175	-1.10914	N	0.2117	-2.4551	-1.1183
N	-2.38676	1.02215	-0.54333	N	2.3949	-1.0090	-0.5185
N	-1.97312	-1.20311	0.93744	N	1.9801	1.2177	0.9721
N	0.53456	-1.56855	1.83364	N	-0.5341	1.5976	1.8631
N	1.75440	-1.63461	2.33365	N	-1.7541	1.6726	2.3618
N	-0.90746	-3.14206	-1.33661	N	0.8922	3.1354	-1.3654
N	0.20099	-2.46194	-1.10883	N	-0.2120	2.4553	-1.1186
N	2.38688	-1.02202	-0.54316	N	-2.3951	1.0087	-0.5191
N	1.97302	1.20327	0.93750	N	-1.9802	-1.2175	0.9721
N	-0.53473	1.56863	1.83356	N	0.5339	-1.5969	1.8636
N	-1.75460	1.63464	2.33351	N	1.7539	-1.6717	2.3623
N	-0.00019	-0.00051	-3.12707	N	0.0009	-0.0006	-3.1545
C	0.41813	-4.74072	-2.49060	C	-0.4503	4.7134	-2.5281
C	-0.81798	-4.27330	-2.02022	C	0.7923	4.2564	-2.0645
C	1.37358	-2.93468	-1.55236	C	-1.3908	2.9171	-1.5569
C	2.57751	-2.13266	-1.24859	C	-2.5895	2.1149	-1.2331
C	3.84101	-2.54659	-1.70576	C	-3.8556	2.5258	-1.6847
H	3.92263	-3.46929	-2.27882	H	-3.9395	3.4439	-2.2646
C	4.93531	-1.75932	-1.41978	C	-4.9486	1.7417	-1.3869
H	5.93101	-2.04413	-1.76554	H	-5.9461	2.0247	-1.7289
C	4.76497	-0.57760	-0.67221	C	-4.7746	0.5646	-0.6335
C	3.45035	-0.25433	-0.25408	C	-3.4583	0.2422	-0.2193
C	5.85485	0.28201	-0.31902	C	-5.8650	-0.2913	-0.2735
H	6.85760	0.02326	-0.66396	H	-6.8682	-0.0309	-0.6158
C	5.65095	1.39184	0.44183	C	-5.6605	-1.3997	0.4886
H	6.48783	2.03535	0.71908	H	-6.4971	-2.0417	0.7702
C	3.23035	0.93160	0.54015	C	-3.2383	-0.9422	0.5778
C	4.33988	1.73456	0.90560	C	-4.3481	-1.7433	0.9469
C	4.09173	2.84239	1.73880	C	-4.0997	-2.8527	1.7779
H	4.91751	3.49031	2.03999	H	-4.9262	-3.4987	2.0812
C	2.81151	3.08130	2.18978	C	-2.8186	-3.0964	2.2232
H	2.57773	3.90189	2.86665	H	-2.5848	-3.9195	2.8969
C	1.77828	2.22863	1.76406	C	-1.7843	-2.2457	1.7958
C	0.40541	2.41244	2.28112	C	-0.4108	-2.4374	2.3087
C	-2.00849	2.55139	3.25742	C	2.0041	-2.5937	3.2817
C	-1.02200	3.45679	3.67111	C	1.0131	-3.4962	3.6917
C	-0.41759	4.74044	-2.49111	C	0.4498	-4.7137	-2.5270
C	0.81848	4.27287	-2.02076	C	-0.7929	-4.2562	-2.0640
C	-1.37325	2.93462	-1.55268	C	1.3905	-2.9174	-1.5561
C	-2.57728	2.13276	-1.24885	C	2.5893	-2.1153	-1.2324
C	-3.84074	2.54677	-1.70607	C	3.8553	-2.5262	-1.6842
H	-3.92226	3.46944	-2.27920	H	3.9392	-3.4444	-2.2639
C	-4.93510	1.75961	-1.42007	C	4.9483	-1.7421	-1.3866
H	-5.93077	2.04448	-1.76588	H	5.9458	-2.0251	-1.7288
C	-4.76488	0.57791	-0.67244	C	4.7744	-0.5649	-0.6334
C	-3.45031	0.25456	-0.25425	C	3.4582	-0.2424	-0.2191
C	-5.85484	-0.28161	-0.31925	C	5.8648	0.2911	-0.2736
H	-6.85755	-0.02279	-0.66423	H	6.8680	0.0307	-0.6161
C	-5.65104	-1.39141	0.44165	C	5.6605	1.3996	0.4884
H	-6.48798	-2.03486	0.71890	H	6.4971	2.0416	0.7698
C	-3.23042	-0.93136	0.54003	C	3.2382	0.9421	0.5778

C	-4.34002	-1.73422	0.90548	C	4.3481	1.7433	0.9468
C	-4.09198	-2.84203	1.73874	C	4.0997	2.8527	1.7777
H	-4.91782	-3.48988	2.03993	H	4.9262	3.4988	2.0808
C	-2.81180	-3.08102	2.18978	C	2.8186	3.0965	2.2230
H	-2.57810	-3.90159	2.86670	H	2.5849	3.9196	2.8967
C	-1.77849	-2.22844	1.76406	C	1.7843	2.2459	1.7957
C	-0.40565	-2.41230	2.28119	C	0.4108	2.4377	2.3086
C	2.00820	-2.55134	3.25760	C	-2.0041	2.5944	3.2816
C	1.02165	-3.45667	3.67129	C	-1.0129	3.4963	3.6920
H	-0.50032	5.66705	-3.06690	H	0.5413	-5.6325	-3.1137
H	1.75317	4.80875	-2.19602	H	-1.7241	-4.7922	-2.2567
H	0.50097	-5.66736	-3.06633	H	-0.5419	5.6320	-3.1150
H	-1.75259	-4.80935	-2.19538	H	1.7234	4.7926	-2.2569
H	3.02297	-2.56496	3.66018	H	-3.0192	2.6152	3.6831
H	1.23032	-4.24264	4.40335	H	-1.2183	4.2869	4.4199
H	-3.02328	2.56497	3.65995	H	3.0191	-2.6144	3.6833
H	-1.23075	4.24277	4.40314	H	1.2188	-4.2871	4.4192
N	-1.52801	4.06896	-2.24520	N	1.5557	-4.0425	-2.2608
N	-0.20224	-3.38303	3.17984	N	0.2110	3.4136	3.2024
N	1.52847	-4.06906	-2.24479	N	-1.5562	4.0419	-2.2619
N	0.20191	3.38319	3.17972	N	-0.2108	-3.4137	3.2021
	**[$EuBTPhen_2$-ClO_4]$^{2+}$**				**[$AmBTPhen_2$-ClO_4]$^{2+}$**		
Eu	0.00001	-0.00002	-0.09603	Am	0.0000	0.0002	0.0787
N	-0.87936	-3.11849	-1.21802	N	0.8674	-3.1201	1.2398
N	0.22727	-2.44609	-0.96045	N	-0.2359	-2.4469	0.9702
N	2.39294	-0.99188	-0.38058	N	-2.4004	-0.9858	0.3660
N	1.94329	1.22575	1.09407	N	-1.9561	1.2375	-1.1144
N	-0.56885	1.54545	1.98572	N	0.5600	1.5750	-2.0061
N	-1.78978	1.58812	2.48542	N	1.7807	1.6291	-2.5057
N	0.87957	3.11820	-1.21844	N	-0.8659	3.1187	1.2451
N	-0.22711	2.44593	-0.96075	N	0.2371	2.4456	0.9739
N	-2.39286	0.99196	-0.38065	N	2.4009	0.9854	0.3657
N	-1.94331	-1.22560	1.09414	N	1.9549	-1.2358	-1.1174
N	0.56881	-1.54531	1.98583	N	-0.5620	-1.5713	-2.0081
N	1.78974	-1.58799	2.48554	N	-1.7828	-1.6243	-2.5072
C	-0.45259	4.65760	-2.44333	C	0.4816	4.6439	2.4712
C	0.78656	4.21208	-1.95817	C	-0.7633	4.2062	1.9932
C	-1.40035	2.89480	-1.42459	C	1.4157	2.8872	1.4303
C	-2.59778	2.09605	-1.09261	C	2.6083	2.0869	1.0836
C	-3.86859	2.49740	-1.53974	C	3.8807	2.4866	1.5268
H	-3.96072	3.41366	-2.12153	H	3.9740	3.3999	2.1128
C	-4.95479	1.70531	-1.23619	C	4.9661	1.6970	1.2160
H	-5.95589	1.98122	-1.57354	H	5.9683	1.9720	1.5509
C	-4.76824	0.52779	-0.48556	C	4.7778	0.5225	0.4618
C	-3.44749	0.21654	-0.07948	C	3.4563	0.2109	0.0575
C	-5.84636	-0.34198	-0.12088	C	5.8575	-0.3436	0.0938
H	-6.85507	-0.09245	-0.45506	H	6.8658	-0.0916	0.4272
C	-5.62399	-1.45136	0.63564	C	5.6369	-1.4525	-0.6634
H	-6.45176	-2.10343	0.92008	H	6.4653	-2.1029	-0.9497
C	-3.20783	-0.96746	0.70995	C	3.2190	-0.9727	-0.7339
C	-4.30487	-1.78300	1.08449	C	4.3176	-1.7861	-1.1093
C	-4.03598	-2.89127	1.91055	C	4.0507	-2.8964	-1.9332
H	-4.85112	-3.54999	2.21722	H	4.8677	-3.5529	-2.2400
C	-2.74871	-3.11670	2.34856	C	2.7637	-3.1277	-2.3680

H	-2.49890	-3.93663	3.02051	H	2.5158	-3.9506	-3.0369
C	-1.72967	-2.25017	1.91741	C	1.7422	-2.2637	-1.9375
C	-0.35249	-2.41259	2.42792	C	0.3650	-2.4355	-2.4454
C	2.06429	-2.50584	3.40210	C	-2.0524	-2.5490	-3.4182
C	1.09863	-3.43712	3.80796	C	-1.0811	-3.4772	-3.8182
C	0.45289	-4.65785	-2.44289	C	-0.4787	-4.6459	2.4666
C	-0.78628	-4.21242	-1.95768	C	0.7656	-4.2080	1.9875
C	1.40053	-2.89489	-1.42433	C	-1.4140	-2.8887	1.4276
C	2.59791	-2.09602	-1.09245	C	-2.6070	-2.0883	1.0828
C	3.86874	-2.49734	-1.53955	C	-3.8790	-2.4889	1.5263
H	3.96091	-3.41364	-2.12127	H	-3.9715	-3.4030	2.1113
C	4.95490	-1.70518	-1.23605	C	-4.9649	-1.6994	1.2170
H	5.95601	-1.98107	-1.57338	H	-5.9668	-1.9752	1.5522
C	4.76830	-0.52762	-0.48551	C	-4.7774	-0.5239	0.4640
C	3.44753	-0.21639	-0.07945	C	-3.4563	-0.2113	0.0594
C	5.84638	0.34223	-0.12089	C	-5.8578	0.3421	0.0975
H	6.85510	0.09271	-0.45505	H	-6.8658	0.0892	0.4312
C	5.62397	1.45164	0.63556	C	-5.6380	1.4519	-0.6585
H	6.45171	2.10377	0.91996	H	-6.4669	2.1022	-0.9437
C	3.20783	0.96764	0.70990	C	-3.2200	0.9734	-0.7307
C	4.30483	1.78325	1.08439	C	-4.3192	1.7866	-1.1047
C	4.03589	2.89157	1.91037	C	-4.0532	2.8980	-1.9275
H	4.85100	3.55034	2.21700	H	-4.8706	3.5543	-2.2331
C	2.74861	3.11697	2.34837	C	-2.7664	3.1304	-2.3626
H	2.49877	3.93695	3.02025	H	-2.5192	3.9543	-3.0306
C	1.72961	2.25037	1.91727	C	-1.7443	2.2665	-1.9334
C	0.35242	2.41279	2.42775	C	-0.3674	2.4396	-2.4416
C	-2.06436	2.50602	3.40192	C	2.0495	2.5553	-3.4154
C	-1.09874	3.43735	3.80773	C	1.0777	3.4838	-3.8135
H	0.53821	-5.55772	-3.05910	H	-0.5709	-5.5406	3.0892
H	-1.71928	-4.74081	-2.16195	H	1.6954	-4.7372	2.2033
H	-0.53788	5.55746	-3.05957	H	0.5744	5.5383	3.0941
H	1.71960	4.74036	-2.16255	H	-1.6929	4.7352	2.2103
H	-3.07909	2.49949	3.80474	H	3.0640	2.5582	-3.8185
H	-1.32554	4.22527	4.53225	H	1.2997	4.2790	-4.5314
H	3.07902	-2.49932	3.80492	H	-3.0671	-2.5511	-3.8208
H	1.32541	-4.22499	4.53254	H	-1.3037	-4.2712	-4.5373
N	-1.56148	4.00100	-2.15807	N	1.5865	3.9870	2.1711
N	-0.12696	-3.38585	3.31824	N	0.1443	-3.4157	-3.3293
N	1.56173	-4.00111	-2.15775	N	-1.5840	-3.9889	2.1680
N	0.12686	3.38609	3.31801	N	-0.1474	3.4212	-3.3241
Cl	-0.00017	-0.00029	-3.25928	Cl	0.0015	-0.0033	3.2945
O	-1.15050	-0.29375	-2.28885	O	1.1650	-0.2519	2.3257
O	-0.30797	1.18787	-4.05395	O	0.2651	1.1940	4.0925
O	1.15028	0.29324	-2.28900	O	-1.1628	0.2486	2.3278
O	0.30742	-1.18854	-4.05390	O	-0.2614	-1.2036	4.0885
		**$Eu(ClO_4)_3$**				**$Am(ClO_4)_3$**	
Eu	0.00130	-0.00007	0.00046	Am	0.0026	0.0000	0.0000
Cl	-1.55091	-2.58146	-0.00020	Cl	3.0693	-0.0003	0.0000
Cl	3.01292	-0.05015	-0.00022	Cl	-1.5377	-2.6517	0.0000
Cl	-1.46389	2.63170	-0.00025	Cl	-1.5371	2.6520	0.0000
O	-1.00208	-3.81786	-0.52517	O	2.0656	-0.5652	-1.0478
O	-2.90037	-2.67574	0.52470	O	2.0657	0.5647	1.0478
O	-1.47139	-1.46054	-1.07421	O	3.8460	-1.0799	0.5811

O	-0.59786	-1.98634	1.07396
O	3.77097	-1.17225	0.52106
O	2.00309	-0.53987	-1.07577
O	3.80756	1.04617	-0.52182
O	2.02040	0.47281	1.07580
O	-2.80987	2.77031	-0.52426
O	-0.87385	3.84966	0.52312
O	-0.53198	2.00417	-1.07448
O	-1.42090	1.50979	1.07484

$Eu(NO_3)_3$

Eu	-0.00010	-0.06271	0.00000
N	2.48715	-1.28426	0.00000
N	0.00196	2.69987	0.00000
N	-2.48889	-1.28121	0.00000
O	1.85835	-0.97892	1.07698
O	1.85835	-0.97892	-1.07698
O	3.55359	-1.80177	0.00000
O	0.00152	2.00069	1.07618
O	0.00152	2.00069	-1.07618
O	0.00271	3.88514	0.00000
O	-1.85981	-0.97644	1.07697
O	-1.85981	-0.97644	-1.07697
O	-3.55580	-1.79776	0.00000

$Eu(ClO_4)_3(H_2O)_4$

O	1.09856	-2.08028	0.39710
H	0.67979	-2.92008	0.14412
H	2.06006	-2.12213	0.21686
O	0.87737	-0.03066	-2.02265
H	1.82765	0.19461	-2.13952
H	0.38004	0.42802	-2.71200
O	-0.10052	-0.12609	2.63646
H	-0.19106	0.76436	3.00514
H	-0.82840	-0.65858	2.98740
O	0.48991	2.16762	1.37743
H	1.42059	2.36361	1.18944
H	-0.03895	2.94560	1.10916
Cl	3.60601	0.07529	-0.21958
O	2.28193	0.54287	0.43354
O	3.50843	0.41927	-1.66783
O	4.70378	0.77627	0.43258
O	3.68886	-1.39868	-0.04055
Cl	-1.98604	-2.39426	-0.17519
O	-3.41857	-2.59450	-0.31334
O	-1.21249	-3.65168	-0.25221
O	-1.44810	-1.41592	-1.22187
O	-1.63762	-1.68004	1.13144
Cl	-2.08656	2.29579	-0.35153
O	-3.32210	2.43388	-1.10137
O	-2.17829	1.21130	0.71462
O	-0.94234	1.78336	-1.23751
O	-1.65099	3.56489	0.28198
Eu	-0.05207	0.00041	0.19848

$Eu(NO_3)_3(H_2O)_4$

O	0.99930	0.21304	-2.38433

O	3.8462	1.0791	-0.5811
O	-1.5095	-1.5073	-1.0557
O	-0.5566	-2.0609	1.0550
O	-2.8679	-2.7763	0.5668
O	-0.9885	-3.8699	-0.5663
O	-0.5561	2.0610	-1.0550
O	-1.5092	1.5077	1.0557
O	-0.9877	3.8701	0.5664
O	-2.8673	2.7769	-0.5668

$Am(NO_3)_3$

N	-2.0800	1.9073	0.0006
O	-1.8876	1.0802	-0.9637
O	-2.9560	2.7048	0.0022
O	-1.2368	1.7912	0.9631
Am	0.0025	0.0030	-0.0008
N	2.7004	0.8302	0.0011
O	2.1729	0.1588	0.9613
O	3.8333	1.1765	0.0017
O	1.8884	1.0920	-0.9596
N	-0.6283	-2.7470	0.0007
O	-0.8944	-3.9014	0.0007
O	-0.9403	-1.9564	0.9643
O	-0.0015	-2.1728	-0.9630

$AmEu(ClO_4)_3(H_2O)_4$

O	-1.1558	2.0877	0.4863
H	-0.7595	2.9411	0.2394
H	-2.1194	2.1111	0.3118
O	-0.8923	-0.0002	-2.0742
H	-1.8472	-0.2165	-2.1799
H	-0.4078	-0.4294	-2.7917
O	0.2569	0.1111	2.6485
H	0.3931	-0.7622	3.0425
H	0.9801	0.6787	2.9530
O	-0.4664	-2.1914	1.3861
H	-1.3983	-2.3967	1.2126
H	0.0636	-2.9650	1.1052
Cl	-3.6302	-0.1171	-0.2374
O	-2.3025	-0.5920	0.4074
O	-3.5231	-0.4157	-1.6956
O	-4.7239	-0.8485	0.3876
O	-3.7288	1.3496	-0.0136
Cl	1.9142	2.5109	-0.2236
O	3.3275	2.7751	-0.4357
O	1.0883	3.7372	-0.2085
O	1.3546	1.5491	-1.2768
O	1.6737	1.7363	1.0744
Cl	2.1209	-2.3418	-0.3840
O	3.3466	-2.5123	-1.1442
O	2.2435	-1.2440	0.6664
O	0.9766	-1.8246	-1.2707
O	1.6689	-3.5941	0.2719
Am	0.0533	-0.0088	0.1768

$Eu(NO_3)_3(H_2O)_4$

O	-2.1982	-2.7897	-1.4995

H	1.77887	0.78561	-2.33769
H	0.34622	0.69928	-2.90848
O	-0.26971	-0.84359	2.21767
H	-1.14456	-1.26818	2.34539
H	-0.26197	-0.05627	2.78153
O	-0.95440	-2.29321	-0.30776
H	-0.27322	-2.97047	-0.18973
H	-1.64181	-2.46986	0.36722
O	-2.71354	-1.91436	1.74016
H	-2.96348	-1.08754	1.26509
H	-3.51570	-2.26421	2.14529
N	2.75395	-1.22630	-0.09718
O	2.57079	0.03364	-0.09990
O	1.68137	-1.91900	-0.07274
O	3.83928	-1.72392	-0.11686
N	0.55509	2.60916	0.77164
O	0.47114	1.61357	1.57189
O	0.41853	2.31232	-0.45575
O	0.74311	3.72321	1.15723
N	-2.59755	0.32151	-0.96612
O	-2.28414	0.21054	0.27902
O	-3.73814	0.37070	-1.32317
O	-1.61537	0.36216	-1.75176
Eu	0.15095	-0.07079	-0.08026
	**BTPhen**		
N	3.22787	-3.24708	-0.30186
N	2.63823	-2.07012	-0.28422
N	1.36996	0.33223	-0.01665
N	-1.36996	0.33226	0.01666
N	-2.63820	-2.07017	0.28385
N	-3.22785	-3.24712	0.30141
C	5.28942	-2.16399	0.16380
C	4.53862	-3.31364	-0.07151
C	3.38365	-0.98431	-0.03346
C	2.69271	0.33468	-0.00419
C	3.47156	1.51381	0.03414
H	4.55760	1.43604	0.05657
C	2.82140	2.72507	0.03814
H	3.38222	3.66276	0.05705
C	1.41263	2.75822	0.01669
C	0.72943	1.51102	0.00025
C	0.68019	3.98721	0.01048
H	1.23721	4.92708	0.02003
C	-0.68012	3.98722	-0.01059
H	-1.23713	4.92710	-0.02016
C	-0.72940	1.51103	-0.00028
C	-1.41258	2.75824	-0.01675
C	-2.82135	2.72511	-0.03819
H	-3.38215	3.66280	-0.05715
C	-3.47153	1.51386	-0.03413
H	-4.55757	1.43608	-0.05650
C	-2.69271	0.33471	0.00423
C	-3.38367	-0.98429	0.03355
C	-4.53867	-3.31363	0.07146

H	-1.6163	-2.3334	-2.1318
H	-2.7827	-2.0604	-1.2244
O	-0.1806	-0.8785	-2.2878
H	0.7544	-1.0998	-2.4412
H	-0.3030	-0.0270	-2.7409
O	-0.5757	-2.3841	0.4713
H	-0.0971	-3.1324	0.8434
H	-1.2139	-2.7237	-0.2315
O	0.3094	0.4893	2.5926
H	1.2205	0.6406	2.8802
H	-0.2227	1.2204	2.9360
N	0.8334	2.5376	-0.9003
O	1.1819	3.5782	-1.3686
O	1.2522	2.1093	0.2235
O	0.0018	1.7633	-1.4935
N	-2.8365	0.4872	0.4482
O	-4.0028	0.6986	0.5898
O	-1.9498	0.9535	1.2363
O	-2.3751	-0.2300	-0.5015
N	2.7217	-1.1703	0.1347
O	2.1033	-0.7704	-0.9137
O	2.0719	-1.0296	1.2169
O	3.8175	-1.6422	0.0875
Am	0.0377	-0.0253	0.1843
		**H_2O**	
O	0.00000	0.00000	0.12055
H	0.00000	0.75321	-0.48219
H	0.00000	-0.75321	-0.48219
		**NO3-**	
N	0.00000	0.00000	0.00000
O	0.00000	1.25000	0.00000
O	1.08253	-0.62500	0.00000
O	-1.08253	-0.62500	0.00000
		**ClO_4^-**	
Cl	0.00000	0.00000	-0.00026
O	0.00000	0.00000	1.49495
O	0.00000	1.40998	-0.49813
O	1.22108	-0.70499	-0.49813
O	-1.22108	-0.70499	-0.49813

C	-5.28952	-2.16392	-0.16336
H	6.37026	-2.20398	0.33592
H	4.98153	-4.31212	-0.08844
H	-4.98158	-4.31211	0.08829
H	-6.37043	-2.20385	-0.33512
N	4.70908	-0.97490	0.18251
N	-4.70917	-0.97484	-0.18202
